# Marriage Law Reform

**Published:** 1921-03-19

**Authors:** 


### MARRIAGE LAW REFORM.

The state of the marriage laws is a question which very closely affects the social and physical well-being of the British people. For a long time past judges, magistrates, health administrators, sociologists, and religious workers who come in contact with every class of society, have recognised the need of a reform which will not only put the sexes on an equality, but will legalise the dissolution of unions which are nothing less than a mockery of marriage and which, besides increasing the sum of human unhappiness, tend definitely to the spread of immorality and the perpetuation of disease. But, in spite of the gradual breaking down of ecclesiastical and sex prejudice, notwithstanding the recommendations of a powerful Divorce Commission, and the persistent advocacy of many disinterested public men, it seems still almost as difficult to get a moderate measure of reform on to the Statute Book as to make the Germans pay. Lord Buckmaster's Bill, carefully designed to abolish the worst abuses of legal restrictions and to meet the objections of those who rightly fear the consequences of the cheapening of the marriage tie, passed the House of Lords but failed to run the gauntlet of the House of Commons. Now Lord Gorell has introduced an emasculated version of the Buckmaster Bill about which all that we can say is that its one merit of establishing women in a position of equality with men in the matter of misconduct as a ground for divorce is quite insufficient compensation for its many important omissions.

The Bill, as it stands, confers no rights in respect of lunacy, habitual drunkenness, desertion, and venereal disease, all of which produce a steadily increasing amount of wretchedness, injustice, and serious temptation to evade or flout the law; and we are in full agreement with the candid and courageous view of the Bishop of Durham that the Bill cannot satisfy the public conscience, and therefore gives no hope of finality or effectiveness. In its present form it will accomplish very little in mitigation of the far-reaching evils of improper marriage relationships, and even Lord Buckmaster's single amendment to meet the case of desertion—a proposal obviously intended to conciliate the waverers rather than put forward as expressing the full convictions of the proposer—is utterly inadequate to satisfy the necessities of the time. The question is one that has to be faced boldly and honestly if the laws of divorce are to be framed in accordance with the demands of justice and the artificial conditions of modern existence. It is not merely that the happiness of a relatively small number of individuals is concerned. The marriage laws as they now operate have created a kind of social cancer in the life of the community. They place the stigma of illegitimacy on thousands of children; they are increasing every day the number of irregular unions; it is not too much to say that they constitute no inconsiderable factor in infant mortality, and they are lowering the standard of morality and decency in every class. No tinkering or timid reform will effect any change worth the effort in these conditions, and a Bill of the Gorell type, unless it is remodelled from top to bottom, may prove more of a curse than a blessing by anæsthetising Parliamentary responsibility.

There is one clause in Lord Gorell's measure which we hope will be retained in its entirety, whatever modifications are suggested for gingering it up into a useful and practical instrument of reform. We refer to Clause 35, which gives the Courts power to exclude all persons "if the interests of decency and justice so require," except the persons directly concerned in the case, "provided that nothing in this sub-section shall authorise the exclusion of *bona-fide* representatives of a newspaper or a news agency." For the first time the Press is thus given a statutory right to representation at all Divorce Court hearings, except those held in *camerâ*; and at the same time there are highly desirable restrictive proposals which prohibit the publication of divorce reports until the conclusion of the proceedings, and make the publication of any pictorial representations of the proceedings contempt of court. These two provisions are conceived, in our opinion, in the interest of the public as well as in the interest of the best elements of the British Press.

